# In utero exposure to the 1918 pandemic influenza in Denmark and risk of dementia

**DOI:** 10.1111/irv.12542

**Published:** 2018-03-01

**Authors:** Noelle M. Cocoros, Anne G. Ording, Erzsébet Horváth‐Puhó, Victor W. Henderson, Henrik T. Sørensen

**Affiliations:** ^1^ Department of Population Medicine Harvard Medical School and Harvard Pilgrim Health Care Institute Boston MA USA; ^2^ Department of Clinical Epidemiology Aarhus University Aarhus Denmark; ^3^ Departments of Health Research & Policy and Neurology & Neurological Sciences Stanford University Stanford CA USA

**Keywords:** Alzheimer's disease, dementia, influenza—human, influenza pandemic—1918–1919

## Abstract

**Background:**

Substantial but inconclusive evidence suggests in utero exposure to influenza infection may be linked with Alzheimer′s disease.

**Objectives:**

We examined whether individuals exposed in utero to the 1918 influenza pandemic are at increased risk of dementia.

**Patients/Methods:**

In this cohort study, surveillance data were used to identify months when influenza activity was at its peak during the pandemic. Using birth dates, exposed and unexposed individuals were identified based on whether they were in utero during ≥1 of the peak months. The outcome, any type of dementia, was identified in population‐based medical registries. Time and age at risk were restricted so exposed and unexposed had equal time at risk; diagnoses for dementia were assessed between ages 62 and 92, with a maximum of 30 years at risk. Poisson regression was used to estimate sex‐adjusted incidence rate ratios (IRRs).

**Results:**

We identified 106 479 exposed and 177 918 unexposed persons. Using the cumulative risk function, there were similar proportions of exposed and unexposed with a dementia diagnosis at 11.9% and 11.7%, respectively. Across all ages, the IRR for the association between in utero influenza exposure and any dementia was 1.01 (95% CI 0.99‐1.04); for Alzheimer's disease, it was 0.97 (0.93‐1.01). When stratified by age and sex, and when dementia type was examined, estimates of association were also null or close to null.

**Conclusions:**

Our study suggests there is likely not an association between in utero exposure to the 1918 influenza pandemic and dementia among those 62 and older.

## 1 INTRODUCTION

Certain prenatal exposures have been associated with adverse health outcomes later in life. The long‐term consequences of in utero exposure to infections, and the impact of exposure to influenza pandemics specifically, have been the source of much work. For example, numerous groups have analyzed the association between in utero exposure to the 1957 influenza pandemic and schizophrenia, although results have been contradictory and clear evidence of an association is lacking.^1^, ^2^, ^3^ The potential effects associated with in utero exposure to the 1918 influenza pandemic have been studied in relation to cardiovascular and other outcomes, with conflicting findings.^4^, ^5^, ^6^

Some investigators have hypothesized that infections may be causally associated with Alzheimer's disease, citing substantial but inconclusive evidence,^7^ with chronic infections in particular receiving more attention.^8^ We assessed whether individuals who were in utero during peaks of the 1918 influenza pandemic—a proxy for in utero influenza exposure—are at increased risk of Alzheimer's disease, vascular dementia, or other forms of dementia, compared to individuals born in the months soon before or after the pandemic peaked.

## 2 METHODS

We used historic Danish surveillance data for influenza‐like illness (ILI) to identify peaks of ILI, as previously reported.^5^ We reviewed historic surveillance data on physician‐reported cases of influenza‐like illness (ILI) in Denmark for the years 1915 through 1922.^9^ The original data contain ILI counts by three broad geographic regions (Copenhagen, “other towns,” and “rural districts”) and by age group. We examined the monthly ILI data and visually identified peaks of activity during the pandemic. The peaks we identified align with those noted in the literature.^10^ We then defined exposed individuals as those who were in utero during at least one of the peak ILI months, based on birth dates and assuming a full 9 months of gestation. Exposed persons had birth dates from November 1918 through October 1920; unexposed persons had birth dates from December 1915 through June 1918 (ie, born before ILI peaks) and March through December 1921 (ie, born after the peaks and were not in utero during any of the ILI peaks).

The primary outcome of interest was dementia of any type which was comprised of Alzheimer's disease, vascular dementia, and other forms of dementia, defined according to diagnostic codes in the *International Classification of Diseases*,* Eighth and Tenth Revisions*.^11^ We also examined Alzheimer's disease, vascular dementia, and other dementia, separately, as secondary outcomes of interest. We used the Danish National Patient Registry (DNPR) and the Psychiatric Central Research Registry (PCRR) 12 to identify exposed and unexposed Danish‐born residents who had an inpatient or outpatient hospital‐based contact for one of the outcomes of interest between January 1, 1977 (when the DNPR was established), and November 30, 2013. The DNPR has recorded information on all admissions to Danish non‐psychiatric hospitals since 1977 and on emergency room and outpatient clinic visits since 1995. The PCRR has recorded psychiatric inpatient diagnoses since 1969 and psychiatric outpatient clinic visits since 1995.^12^, ^13^

Because the DNPR does not include information on diagnoses before 1977, we restricted the time and age ranges during which events could be captured, requiring the exposed and unexposed to have equal time at risk, both in terms of age and years at risk. We excluded individuals with a dementia diagnosis code of interest in the DNPR or PCRR prior to their index date (ie, year they turned 62) as well as those with mild cognitive impairment or amnestic syndrome documented prior to index. Persons born in 1915 (the oldest study individuals) were 62 years old in 1977 when the DNPR was established. Thus, we limit all individuals to those surviving to age 62. Those born in 1921 (the youngest in the study) were 62 years old in 1983 and could be followed until November 30, 2013. Accordingly, the maximum amount of time a person in the 1921 birth cohort could contribute to follow‐up was 30 years (ages 62‐92). We therefore captured diagnoses for dementia occurring between ages 62 and 92, allowing a maximum of 30 years at risk. All persons included in the study were followed from age 62 when they began to contribute follow‐up time until their 92nd birthday, death, dementia diagnosis, emigration, or study end date.

We used Poisson regression to estimate incidence rate ratios (IRRs) and corresponding 95% confidence intervals (CIs) for the outcomes. The incidence rate ratios were adjusted for sex. All analyses were stratified by sex and 10‐year age groups. For a sensitivity analysis, we examined dementia restricted to inpatient diagnoses because only inpatient diagnoses were captured in the DNPR and the PCRR prior to 1995. Cumulative incidence curves were created with death as a competing risk. We conducted an additional sensitivity analysis where the exposed group was limited to those likely exposed during their first trimester, during the first peak of the pandemic (ie, birth dates in April through June 1919), and the unexposed was restricted to those born before June 1918 only (ie, birth dates in December 1915 through May 1918).

We included all persons meeting the study criteria in the analyses. All analyses were conducted using SAS version 9.4 (SAS Institute, Inc., Cary, NC, USA). This study was approved by the Danish Data Protection Agency (record number 2014‐54‐0922).

## 3 RESULTS

We identified 106 479 exposed and 177 918 unexposed persons. There were more females in both groups (52% among both the exposed and unexposed). There were no differences in follow‐up time between the exposed (median years of follow‐up 18.2, interquartile range 11.0‐24.8) and unexposed groups (median years of follow‐up 18.2, interquartile range 11.1‐24.8).

**Figure 1:**
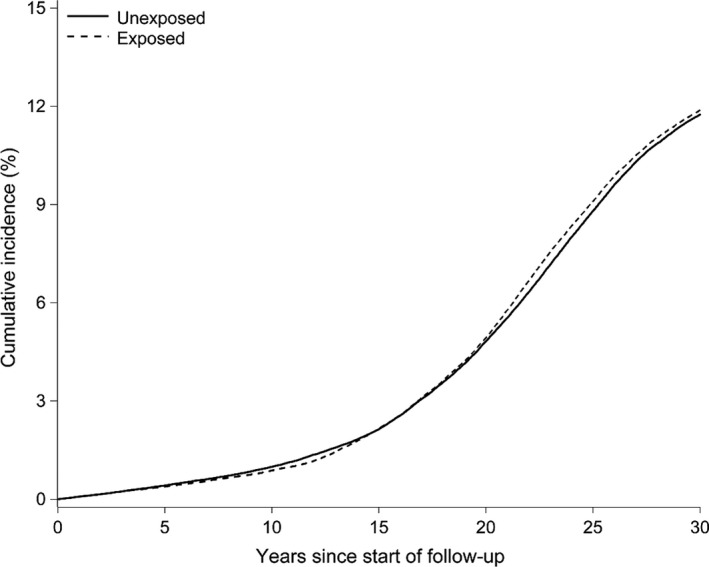
Cumulative incidence of dementia among those exposed and unexposed in utero to the 1918 influenza pandemic in Denmark. Legend: Dementia is defined as any outpatient or inpatient dementia diagnosis

Figure 1 depicts the cumulative incidence of any dementia among all ages under study for the exposed compared to unexposed and shows that the two groups have roughly the same trajectory over time, with the exposed group very slightly diverging with increased risk after 20 years of follow‐up.

There were generally similar proportions of exposed (11.9%) and unexposed (11.7%) individuals with dementia diagnoses during the study period, with more diagnoses among the older age groups (Table 1A). Across all ages and for the three age ranges for which we assessed occurrence of outcomes, we did not observe clear evidence of an association between in utero pandemic influenza exposure and dementia, with the estimates generally being null (Table 1A). For all ages, the IRR for in utero exposure and any dementia (inpatient or outpatient diagnosis) was 1.01 (95% CI 0.99‐1.04); for 62‐72 year olds, IRR 0.89 (95% CI 0.82‐0.96); for >72‐82 year olds, IRR 1.06 (95% CI 1.02‐1.10); and for those older than 82 years, IRR 1.01 (95% CI 0.98‐1.04). The data do not indicate that sex was a modifier of the relationship as female and male strata yielded similar results.

**Table 1:** Incidence rate ratios for in utero exposure to the 1918 influenza epidemic and any dementia diagnosis, (A) outpatient or inpatient setting, in Danish adults by age and (B) inpatient only, in Danish adults by age

			Incidence rate ratios		
	Unexposed (No. with dementia)	Exposed (No. with dementia)	Sex‐adjusted IRR (95% CI)	Female IRR (95% CI)	Male IRR (95% CI)
(A)					
All ages (62‐92 yrs)	177 918 (20 882; 11.7%)	106 479 (12 642; 11.9%)	1.01 (0.99‐1.04)	1.02 (0.99‐1.05)	1.01 (0.97‐1.05)
Ages 62‐72 yrs	177 918 (1761; 0.99%)	106 479 (934; 0.88%)	0.89 (0.82‐0.96)	0.96 (0.86‐1.07)	0.82 (0.74‐0.92)
Ages >72‐82 yrs	138 971 (6817; 4.9%)	83 030 (4308; 5.2%)	1.06 (1.02‐1.10)	1.05 (1.00‐1.11)	1.07 (1.01‐1.13)
Ages >82‐92 yrs	76 716 (12 304; 16.0%)	45 851 (7400; 16.1%)	1.01 (0.98‐1.04)	1.01 (0.97‐1.04)	1.00 (0.95‐1.06)
(B)					
All ages (62‐92 yrs)	177 918 (14 774; 8.3%)	106 479 (8902; 8.4%)	1.01 (0.98‐1.04)	1.02 (0.98‐1.05)	1.00 (0.96‐1.04)
Ages 62‐72 yrs	177 918 (1,750; 0.98%)	106 479 (930; 0.87%)	0.89 (0.82‐0.96)	0.96 (0.86‐1.07)	0.83 (0.74‐0.92)
Ages >72‐82 yrs	138 982 (4641; 3.3%)	83 034 (2816; 3.4%)	1.01 (0.97‐1.06)	1.02 (0.95‐1.08)	1.01 (0.94‐1.09)
Ages >82‐92 yrs	78 148 (8383; 10.7%)	46 817 (5156; 11.0%)	1.03 (0.99‐1.06)	1.02 (0.98‐1.07)	1.03 (0.97‐1.09)

Numbers of exposed and unexposed persons represent those in the study at the start of follow‐up for the age range or calendar period. Crude and sex‐adjusted estimates and confidence intervals were nearly identical, so only sex‐adjusted results are presented. The transition from *International Classification of Diseases*,* Eighth Revision* (ICD‐8), to *Tenth Revision* (ICD‐10) occurred in Denmark in 1995.

IRR, incidence rate ratio; 95% CI, 95% confidence interval.

We also examined whether the association between the exposure and outcome was dependent on type of dementia across all ages (inpatient and outpatient diagnoses). Table 2A shows that the results are null or close to null for all ages combined as well as by age and sex.

**Table 2:** Incidence rate ratios for in utero exposure to the 1918 influenza epidemic and (A) an outpatient or inpatient dementia diagnosis, by type of dementia, in Danish adults and (B) an inpatient dementia diagnosis, by type, in Danish adults

All ages (62‐92 yrs)	Unexposed N = 177 918	Exposed N = 106 479	Incidence rate ratios		
	No. (%) with dementia	No. (%) with dementia	Sex‐adjusted IRR (95% CI)	Female IRR (95% CI)	Male IRR (95% CI)
(A)					
Any dementia	20 882 (11.7)	12 642 (11.9)	1.01 (0.99‐1.04)	1.02 (0.99‐1.05)	1.01 (0.97‐1.05)
Alzheimer's disease	6555 (3.7)	3800 (3.6)	0.97 (0.93‐1.01)	1.01 (0.96‐1.06)	0.91 (0.85‐0.97)
Vascular dementia	2970 (1.7)	1842 (1.7)	1.04 (0.98‐1.10)	1.04 (0.96‐1.12)	1.04 (0.95‐1.14)
Other dementia	11 357 (6.4)	7000 (6.6)	1.03 (1.00‐1.06)	1.02 (0.98‐1.06)	1.06 (1.01‐1.11)
(B)					
Any dementia	14 774 (8.3)	8902 (8.4)	1.01 (0.98‐1.04)	1.02 (0.98‐1.05)	1.00 (0.96‐1.04)
Alzheimer's disease	3667 (2.1)	1976 (1.9)	0.90 (0.85‐ 0.95)	0.96 (0.90‐1.03)	0.82 (0.75‐0.89)
Vascular dementia	1507 (0.85)	919 (0.86)	1.02 (0.94‐ 1.11)	1.04 (0.93‐ 1.16)	0.99 (0.88‐1.12)
Other dementia	9600 (5.4)	6007 (5.6)	1.05 (1.01‐ 1.08)	1.03 (0.99‐1.07)	1.08 (1.02‐1.13)

Numbers of exposed and unexposed persons represent those in the study at the start of follow‐up for the age range. Crude and sex‐adjusted estimates and confidence intervals were nearly identical, so only sex‐adjusted results are presented.

IRR, incidence rate ratio; 95% CI, 95% confidence interval.

As a sensitivity analysis, we restricted dementia diagnoses to those occurring in the inpatient setting. We again see results very consistent with the main analyses where there does not appear to be an association with any dementia (Table 2A) or specific dementia type (Table 2B) and in utero influenza exposure.

The sensitivity analysis restricted to those exposed during their first trimester (during the first ILI peak), compared to those born before June 1918 only, yielded generally similar results compared to the main analysis. However, there were specific age stratifications for particular dementia types with elevated IRRs and statistically significant results, although the point estimates were relatively modest. When collapsed across all ages at risk, any dementia as well as the specific dementia types yielded null results (eg, adjusted IRR for any dementia for all ages 1.02, 95% CI 0.96‐1.08). For the outcome of “other dementia,” the adjusted IRR for ages >72‐82 years was 1.35 (1.18‐1.53). For Alzheimer's disease among those >82‐92 years, the adjusted IRR was 1.19 (1.04‐1.37).

## 4 DISCUSSION

Our study suggests that in utero exposure to the 1918 influenza pandemic is likely not associated with increased risk of dementia after age 62. We observed generally null results but certain stratifications by age, as well as specific dementia types, yielded slightly protective estimates while others suggest slightly increased risk of the outcome, perhaps due to multiple comparisons.

The study was conducted in a large cohort of individuals followed from 1977 through 2013, and the surveillance data used to construct the exposure definition align with multiple other sources. However, exposure misclassification is likely given the approach to defining in utero exposure (eg*,* mothers of children in utero during the ILI peaks may not have been infected with influenza), although such misclassification is likely to be non‐differential and independent.^5^ Healthy survivor bias is a concern from two perspectives. First, we restricted study participation to those who survived to age 62 years. More broadly, the birth rate declined in 1919, with reports of miscarriage and premature birth affecting women pregnant during the pandemic.^10^

The validity of dementia diagnoses varies for different dementia types,^14^ so potential misclassification of dementia subtypes should be considered, as other dementias are known to have lower positive predictive values than diagnoses for Alzheimer′s disease. However, it is unlikely that such misclassification depends on exposure status. Our results are likely generalizable to other western European countries that had similar experiences during the 1918 influenza pandemic, as well as World War I.

There are no studies that we can directly compare our results to. A similar study design was used to examine the association between in utero exposure to pandemics and acute myocardial infarction and stroke in Danish adults, reporting null results for the two outcomes and the 1918 pandemic.^5^ We are not aware of any studies that have examined the 1918 influenza pandemic's association with dementia, although other groups have more broadly studied the role infections may play in Alzheimer's disease. The hypothesized mechanism of actions for such an association includes neuroinflammation and systemic inflammation of the brain.^7^, ^15^

In conclusion, this study does not support the hypothesis that prenatal exposure to the 1918 influenza pandemic is associated with increased risk of dementia at age 62 years or older.

## CONFLICT OF INTEREST

Drs. Cocoros, Ording, Horváth‐Puhó, Henderson, and Sørensen have no disclosures to report. The Department of Clinical Epidemiology is involved in studies with funding from various companies as research grants to (and administered by) Aarhus University. None of these studies have relation to this study.

## AUTHOR CONTRIBUTIONS

NMC led the study design, reviewed and interpreted results, and drafted the draft manuscript; AGO participated in the study design, reviewed and interpreted results, and critically reviewed and approved the final manuscript; EHP participated in the study design, created the dataset, conducted the statistical analysis, assisted in data interpretation, and critically reviewed and approved the final manuscript; VWH participated in the study design, reviewed and interpreted results, and critically reviewed and approved the final manuscript; HTS conceived of the study, participated in the study design, reviewed and interpreted results, and critically reviewed and approved the final manuscript.
